# 7T MRI in the evaluation of ischemic stroke: a systematic review

**DOI:** 10.3389/fnins.2025.1539617

**Published:** 2025-06-23

**Authors:** Felix Tobias Kurz, Daniela Dumitriu La Grange, Daniele Botta, Maria Isabel Vargas, Francois Lazeyras, Myriam Edjlali-Goujon, Jean-Pierre Pruvo, Isabel Wanke, Karl-Olof Lövblad

**Affiliations:** ^1^Division of Neuroradiology, University Hospitals of Geneva, Geneva, Switzerland; ^2^Department of Medical Informatics and Radiology, University of Geneva, Geneva, Switzerland; ^3^Department of Surgery, University of Geneva, Geneva, Switzerland; ^4^Clinique des Grangettes-Hirslanden, Geneva, Switzerland; ^5^Center for Biomedical Imaging of Geneva, Geneva, Switzerland; ^6^Division of Medical Imaging, Raymond Poincaré University Hospital, Garches, France; ^7^Department of Neuroradiology, Lille University Hospital Center, Lille, France; ^8^Division of Neuroradiology, Klinik Hirslanden, Zurich, Switzerland; ^9^Swiss Neuroradiology Institute, Zurich, Switzerland; ^10^Division of Neuroradiology, University of Essen, Essen, Germany

**Keywords:** 7 tesla (7T), ischemic stroke, vessel wall imaging, ultra-high field MRI, neuroimaging

## Abstract

**Introduction:**

7T magnetic resonance imaging (MRI) has advanced in managing neurological and neurovascular diseases. With improved spatial resolution and signal-to-noise ratio, 7T MRI enhances spatial and functional imaging, benefiting ischemic stroke diagnosis, monitoring, and treatment planning.

**Methods:**

To highlight the advances made with ultra-high field MRI in the evaluation of ischemic stroke patients, a systematic review was conducted on the MEDLINE and Web of Science databases using PRISMA guidelines to find peer-reviewed articles from January 1, 1992, to September 1st, 2024. Search terms included “ischemic stroke,” “7T,” “ultra-high field,” “vessel,” “angiography,” and “MRI.” Studies on 7T MRI in adult ischemic stroke patients were included; exclusions were non-human, post-mortem, or pediatric studies.

**Results:**

We identified 16 studies on the use of 7T MRI for prolonged periods after stroke symptom onset, highlighting its higher spatial resolution for depicting ischemic lesions and vascular imaging. Vessel wall imaging (VWI) at 7T was effective for assessing vascular alterations post-thrombectomy and evaluating atherosclerotic lesions, with notable applications in identifying culprit plaques and studying glutamate metabolism changes.

**Conclusion:**

7T MRI advancements open new perspectives for clinical applications and research, particularly in evaluating the impact of thrombectomy strategies and developing treatments to prevent stroke recurrence. Continued research and protocol validation are essential for integrating 7T MRI into routine practice, improving management of neurological and neurovascular diseases.

## 1 Introduction

7T magnetic resonance imaging (MRI) made large strides towards its clinical use in the management of various neurological and neurovascular diseases (Zhang and Shi, 2022; Rados et al., 2022; Shaffer et al., 2022). As it is well-known, ultra-high field MRI provides improved spatial resolution and signal-to-noise ratio compared to lower field, which are key for achieving more precise morphological imaging (Okada et al., 2022), but also for functional and metabolic characterization of cerebral tissue, as well as flow imaging (Shao et al., 2021; Feinberg et al., 2023; Hangel et al., 2022). Ultra-high field MRI’s improved capabilities are particularly beneficial in the context of ischemic stroke, a condition caused by an obstruction in the blood vessels supplying the brain, which requires immediate and accurate diagnosis for effective treatment, but also detailed and robust monitoring following treatment (Salmela et al., 2017; Powers et al., 2019). The superior imaging quality of 7T MRI enables detailed visualization of the brain’s structures, changes in blood flow and tissue blood supply, vascular and microvascular status, as well as tissue diffusive properties, development of tissue necrosis and gliosis, and, eventually, atrophy, all crucial for monitoring ischemic stroke. This precision allows clinicians to identify the affected areas more accurately, to extract morphological or functional post-stroke MRI-based predictors of stroke recurrence and/or stroke outcome (Madai et al., 2012). The high-resolution images facilitate the evaluation of the brain’s response to therapy, enabling healthcare providers to track recovery progress and detect any complications early—this ongoing monitoring is essential for adjusting treatment plans and improving patient outcomes. The ability of 7T MRI to provide detailed and accurate images significantly enhances the effectiveness of both initial assessments and ongoing monitoring of patients, ultimately contributing to better management of neurological and neurovascular diseases.

We review the advances and challenges of ultra-high field MRI in the context of stroke imaging.

## 2 Methods

**Figure 1:**
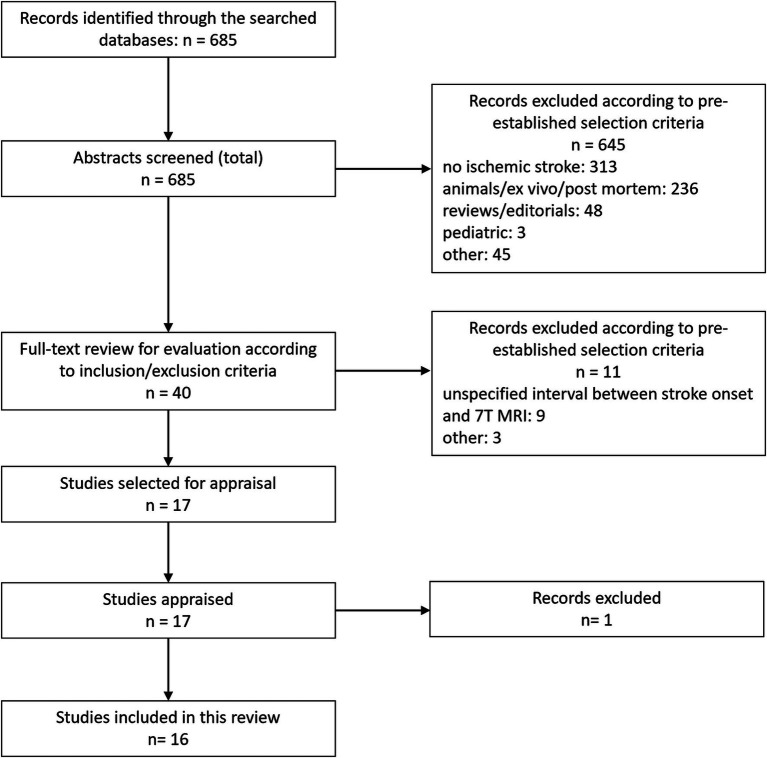
Chart illustrating the study selection process for this review.

This systematic review was performed of the MEDLINE and Web of Science databases according to Preferred Reporting Items for Systematic Reviews and Meta-Analyses guidelines to identify all peer-reviewed articles published in English between January 1, 1992, and September 1st, 2024, using the following predetermined search terms for MEDLINE: “ischemic stroke,” “7T,” “ultra-high field,” “vessel,” “angiography,” “MRI” in the specified combination: (“ischemic stroke”[All fields] OR “angiography”[All fields] OR “vessel”[All fields]) AND (“7T”[All fields] OR “ultra-high field”[All fields]) AND “MRI”[All fields], and (TI = ((“ischemic stroke” OR “stroke” OR “ischemia” OR “apoplexy” OR “ischemic” OR “occlusion” OR “vessel” OR “angiography”) AND (“7Tesla” OR “7T” OR “7T” OR “ultra-high field”) AND (“MRI” OR “magnetic resonance” OR “MR”))) OR AB = ((“ischemic stroke” OR “stroke” OR “ischemia” OR “apoplexy” OR “ischemic” OR “occlusion” OR “vessel” OR “angiography”) AND (“7Tesla” OR “7T” OR “7T” OR “ultra-high field”) AND (“MRI” OR “magnetic resonance” OR “MR”)) for Web of Science. Studies were included if they were original articles describing findings regarding evaluation with 7T MRI of adult patients with ischemic stroke. Studies were excluded if they: did not report findings in human subjects, were ex vivo or post mortem studies on human subjects, did not include patients with an ischemic stroke, did not specific the number of examined stroke patients, did not specify the time interval between stroke onset and 7T MRI exam, did not include exams at 7T MRI, if studies were pediatric studies, or if studies were a case report, see also Figure 1.

Studies were extracted by two authors and reviewed by two authors independently. The present review protocol was unregistered. The last date on which the sources were searched was June 4th, 2024. The quality of the studies included in the review was evaluated using the Newcastle–Ottawa Scale (Stang, 2010).

### 2.1 7T MRI in ischemic stroke

We identified 16 studies reporting the use of MRI at 7T for prolonged time after stroke symptoms onset – within days or several months. These findings are summarized in Table 1.

**Table 1:** Comprehensive list of studies that use 7T brain MRI in ischemic stroke.

Study	Technique/sequences	No of patients	Timing of MRI evaluation	Findings
Kang et al. (2010)	MRA/TOF	10 patients (and 10 age-matched controls)	Within 24 months from stroke onset	Stroke patients had significantly fewer arterial branches, despite no large-vessel abnormalities
Madai et al. (2012)	T1w 3D MPRAGE 2D FLAIR 2D T2 TSE T2*w 2D FLASH MRA/3D-TOF	298	>13 days from stroke onset	Clear benefit in anatomical resolution Constraints related to time scan and brain coverage
Harteveld et al. (2017)	VWI (pre and post contrast)/3D whole-brain MPIR-TSE	25 patients (and 25 matched healthy controls)	Within 3 months from stroke onset	An association between posterior circulation lesion burden/enhancement and ischemic events
Geurts et al. (2019)	2D phase contrast MRI	10 patients (plus 11 patients with deep intracerebral hemorrhage and 18 healthy controls)	Within 48 months from stroke onset	Blood flow pulsatility in perforating arteries is increased and detectable perforators reduced in stroke patients
Truong et al. (2019)	VWI (pre and post contrast)/3D whole-brain MPIR-TSE 3D TFE	7	Within 2 days from treatment	Vessel wall contrast enhancement corresponds to the deployment location of stent retriever
Lindenholz et al. (2020a)	VWI (pre and post contrast)/3D whole-brain MPIR-TSE MRA/TOF	49	Within 3 months from stroke onset	Higher contrast-to-noise ratio and image quality compared to 3 T Changes in the vessel wall following thromboaspiration (enhancing foci)
Lindenholz et al. (2020b)	VWI (pre and post contrast)/3D MPIR-TSE (whole brain and lower FOV) DWI T2w FLAIR MRA/TOF	90 (ischemic stroke and TIA)	Within 3 months from stroke onset	Cardiovascular risks factors are associated with a higher number of vessel wall lesions in anterior circulation
Lindenholz et al. (2021)	VWI (pre and post contrast)/3D MPIR-TSE (whole brain and lower FOV) DWI T2w FLAIR MRA/TOF	82 (ischemic stroke and TIA)	Within 3 months from stroke onset	Associations between the vessel wall lesions in large and small intracranial arteries
Fakih et al. (2020)	HR-VWI (pre and post contrast)/3D TI CUBE T2w CUBE 3D TOF 3D SWAN	38 (Cryptogenic stroke patients)	In average (5.7 months from stroke onset)	Identification of underlying intracranial atherosclerosis disease
Mori et al. (2019)	HR-MRA/CFD 3D FLAIR 3D T2*w	51 (AIS confined in the basal ganglia and/or corona radiata)	Within 3 weeks from stroke onset	Fluid dynamics modifications are risk factors for lateral striate arteries territory infarction
Miyazawa et al. (2019)	HR-MRA	34 (AIS within the lenticulostriate arteries territory)	Within 5–21 days from stroke onset	Occlusive changes in the lenticulostriate arteries territory
Suzuki et al. (2021)	HR-MRA	39 (acute infarcts confined within the lenticulostriate arteries territory)	Within 2 weeks from stroke onset and at one months after onset	Recanalization of the relevant lenticulostriate arteries can occur
Nicolo et al. (2021)	MRS GluCEST	19	2–13 days from stroke onset	Highest ratios between the ipsilateral and contralateral to stroke GluCEST contrast were seen in patients with more severe strokes (consistent with dysregulation of glutamate homeostasis)
Chu et al. (2023)	fMRI (T2*w EPI) during visually cue motor movement	15 patients with TIA (and 28 healthy controls)	Within 25 days from TIA symptoms	Patients with TIA have abnormal motor network connectivity during hand movement tasks
Lucci et al. (2024)	3D T1 MPIR-TSE	78	6–41 days from stroke onset	The number of vessel wall lesions was associated with the severity of intracranial internal carotid artery calcifications
Bai et al. (2024)	3D TOF-MRA 3D T1 VW-MRI	60	Within 4 weeks from stroke onset	Plaque irregular surface and lenticulostriate artery origin involvement accurately predict culprit plaques

2D FLAIR = T2-weighted 2D Fluid Attenuated Inversion Recovery. 2D FLASH = T2* weighted 2D Fast Low Angle Shot Gradient Echo (corresponds to FFE or SPGR from other vendors). 2D T2 TSE = T2-weighted 2D Turbo Spin Echo. Fast Spin Echo (corresponds to T2 FSE from other vendors). 3D TI CUBE = 3-dimensional T1-weighted fast-spin-echo (corresponds to MPIR-TSE or 3D VISTA from other vendors). 3D TFE = 3D T1 weighted fast gradient echo sequence (corresponds to T1 MPRAGE or BRAVO from other vendors). 3D TOF = 3D Time-of-Flight angiography. AIS = acute ischemic stroke; CFD = computational fluid dynamics; DWI = diffusion-weighted imaging; EPI = echo planar imaging; FOV = field of view; Gd = Gadolinium administration; GluCEST = glutamate weighted chemical exchange saturation transfer; HR = high resolution; ICAD = intracranial atherosclerotic disease; MPRAGE = Magnetization-Prepared Rapid-Acquired Gradient-Echo (corresponds to TFE or BRAVO from other vendors); MPIR-TSE = magnetization-prepared inversion recovery turbo spin echo (corresponds to 3D VISTA with DIR preparation or CUBE T1 black-blood with DANTE preparation from other vendors); MRA = magnetic resonance angiography; MRS = magnetic resonance spectroscopy; SWAN = 3-dimensional susceptibility-weighted angiogram (corresponds to SWI or SWIp from other vendors); T1w = T1 weighted, T2w = T2 weighted, T2w = T2 weighted; TIA = transient ischemic attack; VWI = vessel wall imaging. Where appropriate, corresponding sequence names from other vendors are provided.

The improved performance of 7T MRI arises not only from the higher magnetic field strength, but also from changes in intrinsic MRI tissue parameters: 7T MRI offers approximately a 3-4-fold increase in SNR over 3 T in the human brain, particularly in gray and white matter (Pohmann et al., 2016). In addition, longitudinal relaxation times (T1) increase substantially with field strength, whereas transverse relaxation times (T2, T2*) decrease, which has direct implications for optimizing contrast in both structural and functional sequences. These biophysical changes enhance tissue contrast in susceptibility-weighted and T1-weighted imaging, critical for detecting microvascular pathology and cortical/subcortical infarcts in stroke populations.

**Figure 2:**
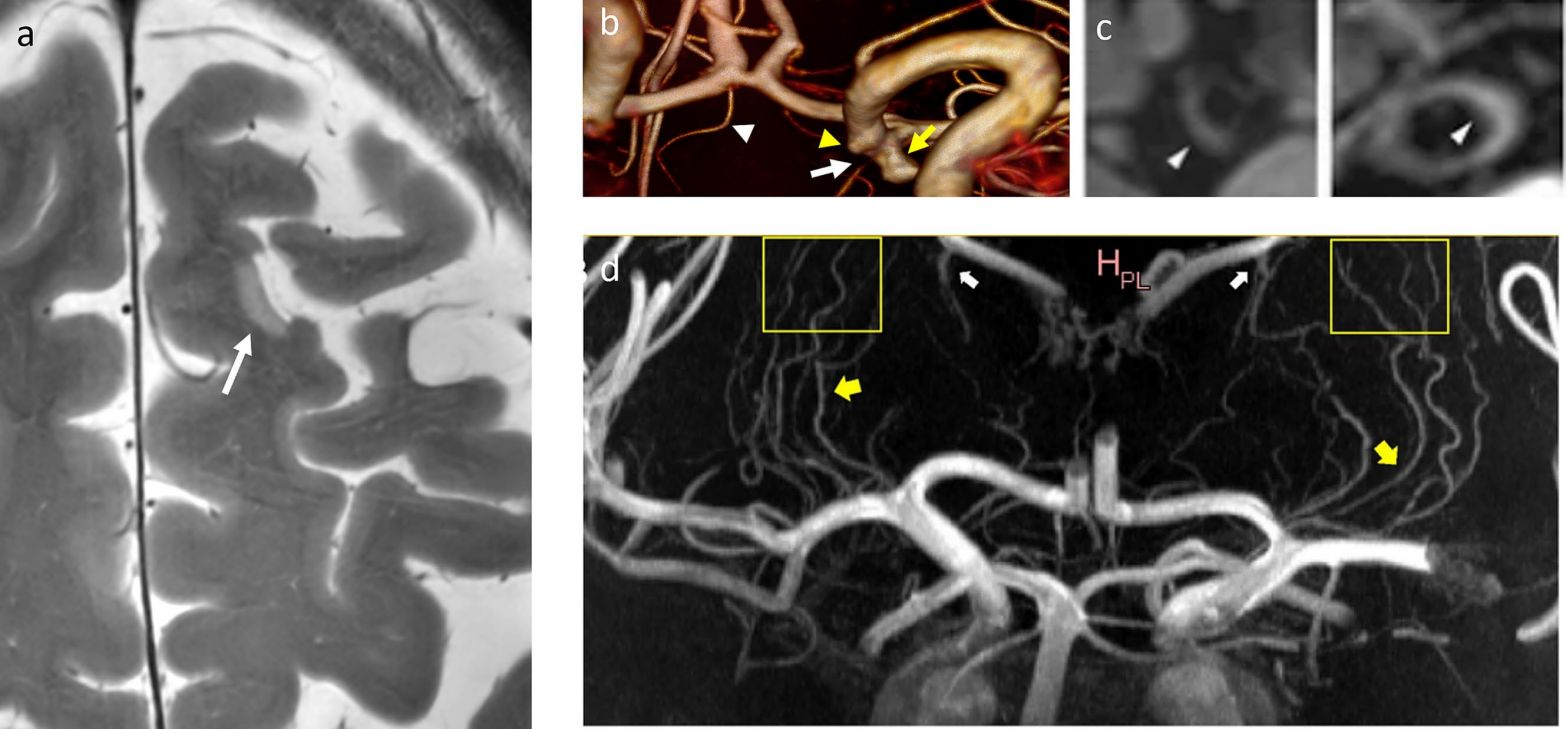
Microinfarcts, lenticulostriate arteries, and vessel wall lesions. **(a)** T2w-hyperintense cortical microinfarction in the left superior frontal gyrus, in-plane-resolution: 0.2 mm x 0.2 mm. **(b)** 3D reconstruction of a 3D TOF MRA indicating the origin of the left choroidal artery (white arrow) from the neck of an aneurysm of the terminal carotid artery (yellow arrow). White arrowhead: right recurrent artery of Heubner, yellow arrowhead: infundibular origin of the left posterior communicating artery. **(c)** Vessel wall lesions and calcifications. From left to right: CT images of a thick calcification in the right ICA and two calcified spots in the left VA, and 7T MR images of an elongated vessel wall lesion in the right ICA and a thicker vessel wall lesion in the left VA, adapted from reference (Lucci et al., 2024) (CC BY 4.0 license). Both ICA and VA exhibit a significant presence of calcifications and vessel wall lesions as opposed to other intracranial arteries. **(d)** Maximum Intensity Projection of a contrast-enhanced TOF-MRA demonstrates lenticulostriate arteries (yellow arrows), that can be followed over long trajectories (yellow boxes), as well as prominent choroidal and thalamostriate veins (white arrows), adapted from reference (Bai et al., 2024) (CC BY 4.0 license). ICA: internal carotid artery, VA: vertebral artery, TOF-MRA: time-of-flight-magnetic resonance angiography.

More than a decade ago, a first study by Kang et al. utilized 7T arterial Time-of-Flight (TOF) magnetic resonance angiography (MRA) to visualize the lenticulostriate arteries in patients with lacunar strokes of the basal ganglia, demonstrating a significantly reduce number of arterial branches and no large-vessel abnormalities when compared to age-matched healthy controls (Kang et al., 2010). A clinically feasible stroke imaging protocol at 7T MRI was proposed and tested in subacute and chronic stroke patients by Madai et al. (2012). This imaging protocol included T1-weighted 3D Magnetization-Prepared Rapid-Acquired Gradient-Echo (3D-MPRAGE), T2-weighted 2D Fluid Attenuated Inversion Recovery (2D-FLAIR), T2-weighted 2D Turbo Spin Echo (2D-T2-TSE), T2*-weighted 2D Fast Low Angle Shot Gradient Echo (2D-HemoFLASH), and 3D arterial TOF MRA. The demonstrated advantage of 7T was the higher spatial resolution when depicting ischemic lesions, periinfarct alterations, and in vessel imaging, see also Figure 2. The pitfalls at the time were prolonged scan time and reduced brain coverage for the 2D FLAIR, 2D T2 TSE, and 3D TOF sequence.

Following its first description by van der Kolk et al. (2013) at 7T, vessel wall imaging (VWI) was effective for depicting vascular alterations associated with mechanical thrombectomy, with thromboaspiration, or with a stent retriever (Truong et al., 2019; Lindenholz et al., 2020a). 7T vessel wall imaging was also used in several studies for evaluating the extent and distribution of atherosclerotic lesions in patients who suffered ischemic stroke or transient ischemic attacks (TIA), the associations between cardiovascular risks factors and vessel wall lesions extent, and identification of atherosclerotic disease as potential underlying cause in cryptogenic stroke (Harteveld et al., 2017; Lindenholz et al., 2020b; Lindenholz et al., 2021; Fakih et al., 2020). Furthermore, VWI could be used to study the association of intracranial arterial calcifications and the presence of vessel wall lesions with vessel types: only the intracranial carotid artery and the vertebral arteries showed a significant presence of both calcifications and vessel wall lesions, whereas other vessels predominantly exhibited vessel wall lesions, see also Figure 2b (Lucci et al., 2024).

MRA at 7T proved useful for evaluating the occlusion status of lenticulostriate arteries and changes in hemodynamic parameters associated with infarction (Mori et al., 2019; Miyazawa et al., 2019; Suzuki et al., 2021). Use of contrast can improve the visualization of cerebral small vessel disease, which can be an underlying condition of ischemic stroke, see Figure 2c (Osuafor et al., 2022). Two-dimensional phase contrast imaging at 7T was used to reveal increased blood flow pulsatility in small vessel disease stroke patients versus healthy controls (Geurts et al., 2019). Recently, VWI at 7T was used to identify culprit plaques in subcortical infarction, using lenticulostriate artery origin involvement and plaque (irregular) surface morphology as independent predictors (Bai et al., 2024).

Changes in glutamate concentration distribution in the brain following ischemic events, were investigated with magnetic resonance spectroscopy (MRS) and glutamate weighted chemical exchange saturation transfer (GluCEST) at 7T (Nicolo et al., 2021). Glutamate metabolism is important for understanding the development of neuronal damage and for devising post-stroke neuroprotective therapies (Nicolo et al., 2019; Shen et al., 2022; Kaplan-Arabaci et al., 2022). Furthermore, functional imaging at 7T revealed altered effective connectivity within motor networks during voluntary movement in patients with transient ischemic attacks, suggesting persistent motor system disruption despite clinical recovery (Chu et al., 2023).

The aforementioned studies, leveraging the advantages of 7T MRI to image ischemic stroke, open up new perspectives for future applications. With the accessibility of 7T MRI in clinical settings, randomized clinical trials aiming to evaluate the impact of thrombectomy devices and strategies on vascular integrity, as well as their implications for functional recovery post-treatment, can greatly benefit from advanced vessel wall imaging. As 7T MRI becomes more feasible in clinical practice, it holds significant potential as a valuable tool for elucidating the underlying vascular pathologies associated with the risk of ischemic stroke. This understanding is crucial for developing treatments to prevent stroke recurrence. Typical parameters for sequences in a 7T MRI stroke protocol are given in Table 2, in comparison to 3 T MRI.

**Table 2:** Typical parameters for sequences in a stroke protocol at 7T MRI and 3 T MRI, see also Zhang and Shi (2022).

Sequence/orientation	Accel meth	TE (in ms)	TR (in ms)	Interslice distance (%)	Slice thickness (in mm)	Voxel size (mm)	Acq time (in min)
7T MRI							
DWI/ax	SMS 3	46, 72	6,200	20	2.0	1.2×1.2×2.0	3
FLAIR/ax	GRAPPA 3	90	11,000	20	2.0	0.6×0.6×1.5	4
T2w/ax	GRAPPA 3	61	3,900	30	3.0	0.1×0.1×3.0	6
T2*w/ax	GRAPPA 3	20	1,080	0	1.5	0.2×0.2×1.5	9
TOF/ax	GRAPPA 2	3.6	20	0	0.4	0.3×0.3×0.4	7
T1w/ax	GRAPPA 3	2.49	3,000	50	0.3	0.3×0.3×0.3	6
VWI/ax	GRAPPA 3	20	1,000	0	0.4	0.4×0.4×0.4	8
3T MRI							
DWI/ax	SMS 3	46,72	6,200	20	2.0	1.2×1.2×2.0	3
FLAIR/ax	GRAPPA 3	74	9,000	20	2.0	0.7×0.7×2.0	3–4
T2w/ax	GRAPPA 3	95–105	4,000–5,000	10–30	3.0	0.9×0.9×3.0	4
T2*w/ax	GRAPPA 2–3	15–25	700–1,000	0	1.5	0.4×0.4×1.5	5
TOF/ax	GRAPPA 2	3.6	22	0	0.5	0.4×0.4×0.5	7
T1w/ax	GRAPPA 2–3	2.5	2,100	50	1.0	1.0×1.0×1.0	5
VWI/ax	GRAPPA 2–3	20	1,000	0	0.6	0.6×0.6×0.6	8

Accel meth: acceleration method, TE: echo time, TR: repetition time, BW: band width, Acq: acquisition, ax: axial, sag: sagittal, cor: coronal, DWI: diffusion weighted imaging, SMS: simultaneous multi slice, FLAIR: fluid attenuating inversion recovery, GRAPPA: generalized autocalibrating partially parallel acquisitions, T2w: T2 weighted, T2*w: T2* weighted, TOF: time-of-flight, T1w: T1 weighted, VWI: vessel-wall imaging.

### 2.2 Perspectives for 7T MRI in the evaluation of acute ischemic stroke

A short duration of the MRI stroke protocol is paramount in the emergency setting, in which clinical decisions must be taken when stroke patients arrive at the hospital. Current clinical stroke protocols at 3 T MRI machines typically include the following sequences: FLAIR, diffusion-weighted imaging (DWI), T1-weighted (T1w) and T2-weighted (T2w) imaging, susceptibility-weighted imaging (SWI), time-of-flight (TOF) angiography, perfusion-weighted MRI (PWI), as well as contrast agent based MR angiography (Fiebach and Galinovic, 2014; Shafaat and Sotoudeh, n.d.). Some centers also add fat-saturated T1w images before contrast administration to screen for hemorrhagic plaques in cervical and/or intracranial dissection. While acute stroke lesions appear within 30 min on DWI images, MR signal changes in the FLAIR sequence occur beyond 3 h from stroke onset, which is a useful feature when combined with information from DWI for estimating the time from onset of stroke (Yoshimoto et al., 2019). The diffusion–perfusion mismatch (ischemic core-to-penumbra ratio) is particularly useful in predicting the response to treatment in patients beyond the early time window (Campbell et al., 2019).

TOF or contrast-agent based MRA can be performed to assess large vessel occlusion, and in addition collaterality. SWI sequences are typically used to rule out intracranial hemorrhage before IV alteplase administration, and to assess the presence or absence of a susceptibility vessel sign (SVS), which is indicative of clot composition, clot vulnerability to treatment, and stroke etiology, and has a predictive value for treatment efficacy (Dumitriu LaGrange et al., 2023a, 2023b). The presence of SVS is associated with red blood cells (RBC)-rich clots, is a predictor of excellent outcome in patients treated with IV alteplase and is associated with successful reperfusion after mechanical thrombectomy; clots which do not display SVS are rich in fibrin/platelets, less likely to be retrieved by aspiration alone and more often require the use of combined therapy. Studies show that quantitative T2* mapping could predict the red blood cells content of the clot (Bourcier et al., 2017; Gilbert et al., 2022), an effect that would be greatly enhanced at 7T MRI.

First centers have begun to incorporate 7T MRI in clinical routine imaging, using dedicated MRI protocols that include 3D T1w magnetization-prepared rapid gradient echo, fat saturated FLAIR, T2w turbo-spin-echo, SWI, and DWI sequences (Özütemiz et al., 2023). However, there are currently no 7T MRI protocols in place for the evaluation of patients with hyperacute or acute ischemic stroke (Vachha and Huang, 2021).

One major challenge are field inhomogeneities (B0 and B1), which can lead to artifacts and signal dropouts, especially in sequences like FLAIR and DWI, which are essential in stroke evaluation. 3D FLAIR brain imaging at 7T can be improved using direct signal control and dedicated parallel transmission coils, which help overcoming the disadvantage of spatial variations caused by field inhomogeneities to allow whole brain imaging (Beqiri et al., 2018). Undoubtedly, FLAIR at 7T brings the advantage of better structural depiction, compared with lower field strengths (Regnery et al., 2019). In addition, the FLAIR sequence detects subarachnoid hemorrhage with high sensitivity and gives a high signal in sulci in patients with subarachnoid bleeding (Shafaat and Sotoudeh, n.d.). There is first evidence that subarachnoid cerebrospinal fluid hyperintensities at 7T FLAIR MRI in cerebral amyloid angiopathy, not always or only retrospectively observed at 3 T MRI, are associated with cortical superficial siderosis and are hypothesized to represent subtle plasma protein or blood residue leakage into the cerebrospinal fluid (Koemans et al., 2023). The specific absorption rate (SAR) and scan time can be reduced for FLAIR at 7T without compromising image quality by scaling the inversion pulse power slice-by-slice (Abbasi-Rad et al., 2021).

DWI at 7T can provide much better contrast, resolution, and SNR, compared to lower field strengths, and developments in reconstruction techniques can further improve the image quality, such as 3D multi-lab acquisition, slab-to-slab phase correction, nonlinear registration for slab alignment, or the use of parallel imaging techniques (Wu et al., 2016; Varela-Mattatall et al., 2023). Another reconstruction framework was introduced recently combining a two-stage N/2 ghost correction approach with an adapted L1-SPIRiT method (Pan et al., 2023). Advances in DWI pulse sequences saw the development of simultaneous multi-slice (SMS) or multiband DWI at 7T (Wu et al., 2018). SMS RESOLVE is commercially available (Siemens Healthineers), and provides distortion free DWI in up to 60% shorter time (Siemens Healthineers, 2025; Siemens Healthineers, n.d.). Still, although in-plane-resolution increases up to 1-2 mm for DWI sequences at 7T, imaging in acute stroke suffers from typical field inhomogeneities at 7T that may be prevent an accurate assessment of small hyperintensities in the b1000 image in a clinical routine setting (Özütemiz et al., 2023). In addition, strong magnetic field gradients of the newest generation of 7T MRI machines may not be fully harnessed due to heart and nerve stimulation limits (Tan et al., 2020; Klein et al., 2021).

The increase in in-plane-resolution up to 0.1–0.2 mm for SWI and T2w sequences allows a much better depiction of cortical lesions (see also Figure 2a), as well as the detection of tiny clots and/or stenoses in peripheral artery branches. Differentiation between blood residues, clots, and calcifications may be achieved at high resolutions with quantitative susceptibility mapping (QSM) at 7T, or in half of the scan time with equal image quality when compared to 3 T MRI, and good reproducibility (Spincemaille et al., 2020).

7T TOF angiography allows assessment of lenticulostriate arteries, anterior and posterior choroidal arteries, as well as peripheral cerebral arteries not before possible at 3 T, to accurately describe intracranial atherosclerotic disease. Presence and variations of anterior and posterior choroidal arteries may allow for an assessment of the hippocampal vascular reserve, that is decisive in developing cognitive dysfunction (Perosa et al., 2020). Small stenoses, but also aneurysms and their vascular connections are much easier to assess with TOF-MRA at 7T MRI (Leemans et al., 2020).

The advantages of vessel-wall imaging at ultra-high field are given for an improved the assessment of intracranial atherosclerosis (see also Figure 2b) and enhanced visualization of aneurysm walls, it may be used in combination with contrast agent administration to assess small and medium vessel vasculitis and dissection (Özütemiz et al., 2023; Feng et al., 2022).

SAR constraints can limit the use of certain sequences or prolong scan times, reducing patient throughput. At 7T MRI, SAR levels increase disproportionately due to the quadratic relationship between field strength and radiofrequency power deposition, presenting a significant challenge for clinical protocols (Markenroth Bloch and Poser, 2021). SAR constraints limit the use of sequences that require high RF power, such as fast spin echo, magnetization transfer imaging, and certain contrast-enhanced sequences. To stay within safety limits, these sequences often require longer repetition times or reduced flip angles, which can lead to longer scan times, lower contrast-to-noise ratios, and reduced temporal efficiency. Scan efficiency is reduced when SAR limits force delays between acquisitions or prevent the use of certain advanced pulse sequences altogether. This can affect patient throughput and workflow in clinical settings, making 7T MRI less practical for time-sensitive evaluations, such as acute stroke imaging. To address these challenges, advanced techniques like parallel transmission (pTx) and B1 shimming have been developed (Deniz, 2019). These methods aim to optimize the RF field distribution, improving homogeneity and reducing local SAR hotspots (Yetisir et al., 2022). For instance, pTx allows for more uniform excitation by adjusting the phase and amplitude of RF pulses across multiple transmit channels, which can help in managing SAR levels effectively. However, implementing these techniques requires specialized hardware and complex calibration processes, which are not yet standard in all clinical settings, e.g., through personalized local SAR predictions (Brink et al., 2022).

Implant compatibility remains a major barrier: many pacemakers, aneurysm clips, and other metallic implants are not approved for 7T imaging. The use of 7T MRI in a clinical emergency setting is therefore still far from given and requires, among other things, the cooperation of the material manufacturers with corresponding safety certifications for high-field examinations that are based on rigorous tests. However, clinical use for post-stroke monitoring is now possible in a few centers for patients with appropriate eligibility, and is and has already been carried out there (Özütemiz et al., 2023). Patient discomfort is another consideration, as 7T MRI can increase dizziness, nausea, and headaches, peripheral nerve stimulation, and claustrophobia (Hansson et al., 2020).

In addition, the major advantages of the higher resolution, particularly in the assessment of vessels and clots, as well as microinfarcts and possibly microscopically small hemorrhages, must be clinically evaluated in larger clinical cohorts on 7T MRI with regard to patient outcome and treatment decisions. The protocols required for this must be validated on a multicenter basis—their creation will pave the way for highly precise clinical diagnosis of ischemic stroke. The high cost and limited availability of 7T systems, however, restrict widespread clinical use, with only a small number of centers worldwide equipped and certified to use them in patients. Inter-study variability due to differences in imaging protocols, coil design, and reconstruction methods remains a challenge for standardization and cross-center reproducibility.

Addressing these technical, logistical, and safety issues through ongoing engineering advances and harmonization initiatives using standardized protocols, cost-effectiveness studies, and large-scale multicenter trials will be essential for broader clinical translation.

The development of MRI systems beyond 7T, such as the 11.7T Iseult system and proposed 14 T human MRI scanners may enable the detection of even smaller microvascular changes and subtle ischemic lesions; however, apart from overcoming technical challenges, a clinical translation will need a thorough assessment of safety profiles first, before potential diagnostic benefits may be validated.

## 3 Conclusion

7T magnetic resonance imaging (MRI) represents a significant advancement in the clinical management of neurological and neurovascular diseases, particularly ischemic stroke. Its ultra-high field strength offers superior spatial resolution and signal-to-noise ratio, enabling detailed visualization of brain structures, blood flow changes, vascular status, and tissue properties. These capabilities are crucial for accurate diagnosis, monitoring, and treatment planning. As 7T MRI becomes more integrated into clinical practice, it holds great potential for improving patient outcomes through enhanced diagnostic accuracy and better-informed treatment decisions. Continued research and multicenter validation of 7T MRI protocols will be essential for fully realizing its clinical benefits in the diagnosis and management of ischemic stroke.

## References

[ref1] Abbasi-Rad S. O’Brien K. Kelly S. Vegh V. Rodell A. Tesiram Y.. (2021). Improving FLAIR SAR efficiency at 7T by adaptive tailoring of adiabatic pulse power through deep learning estimation. Magnetic Res. Med. 85, 2462–2476. doi: 10.1002/mrm.28590, PMID: 33226685

[ref2] Bai X. Fan P. Li Z. Mossa-Basha M. Ju Y. Zhao X.. (2024). Evaluating middle cerebral artery plaque characteristics and lenticulostriate artery morphology associated with subcortical infarctions at 7T MRI. J. Magn. Reson. Imaging 59, 1045–1055. doi: 10.1002/jmri.28839 37259904

[ref3] Beqiri A. Hoogduin H. Sbrizzi A. Hajnal J. V. Malik S. J. (2018). Whole-brain 3DFLAIR at 7T using direct signal control. Magn. Reson. Med. 80, 1533–1545. doi: 10.1002/mrm.27149 29476551 PMC6120540

[ref4] Bourcier R. Brecheteau N. Costalat V. Daumas-Duport B. Guyomarch-Delasalle B. Desal H.. (2017). MRI quantitative T2* mapping on thrombus to predict recanalization after endovascular treatment for acute anterior ischemic stroke. J. Neuroradiol. 44, 241–246. doi: 10.1016/j.neurad.2017.03.006, PMID: 28478114

[ref5] Brink W. M. Yousefi S. Bhatnagar P. Remis R. F. Staring M. Webb A. G. (2022). Personalized local SAR prediction for parallel transmit neuroimaging at 7T from a single T1-weighted dataset. Magn. Reson. Med. 88, 464–475. doi: 10.1002/mrm.29215, PMID: 35344602 PMC9314883

[ref6] Campbell B. C. V. Ma H. Ringleb P. A. Parsons M. W. Churilov L. Bendszus M.. (2019). Extending thrombolysis to 4·5-9 h and wake-up stroke using perfusion imaging: a systematic review and meta-analysis of individual patient data. Lancet 394, 139–147. doi: 10.1016/S0140-6736(19)31053-0 31128925

[ref7] Chu T. Lee S. Jung I. Y. Song Y. Kim H. A. Shin J. W.. (2023). Task-residual effective connectivity of motor network in transient ischemic attack. Commun. Biol. 6:843. doi: 10.1038/s42003-023-05212-3, PMID: 37580508 PMC10425379

[ref8] Deniz C. M. (2019). Parallel transmission for ultrahigh field MRI. Top. Magn. Reson. Imaging 28, 159–171. doi: 10.1097/RMR.0000000000000204, PMID: 31188274 PMC7039313

[ref9] Dumitriu LaGrange D. Hofmeister J. Rosi A. Vargas M. I. Wanke I. Machi P.. (2023a). Predictive value of clot imaging in acute ischemic stroke: a systematic review of artificial intelligence and conventional studies. Neurosci. Inform. 3:100114. doi: 10.1016/j.neuri.2022.100114

[ref10] Dumitriu LaGrange D. Reymond P. Brina O. Zboray R. Neels A. Wanke I.. (2023b). Spatial heterogeneity of occlusive thrombus in acute ischemic stroke: a systematic review. J. Neuroradiol. 50, 352–360. doi: 10.1016/j.neurad.2023.01.004 36649796

[ref11] Fakih R. Roa J. A. Bathla G. Olalde H. Varon A. Ortega-Gutierrez S.. (2020). Detection and quantification of symptomatic atherosclerotic plaques with high-resolution imaging in cryptogenic stroke. Stroke 51, 3623–3631. doi: 10.1161/STROKEAHA.120.031167 32998652

[ref12] Feinberg D. A. Beckett A. J. S. Vu A. T. Stockmann J. Huber L. Ma S.. (2023). Next-generation MRI scanner designed for ultra-high-resolution human brain imaging at 7Tesla. Nat. Methods 20, 2048–2057. doi: 10.1038/s41592-023-02068-7, PMID: 38012321 PMC10703687

[ref13] Feng J. Liu X. Zhang Z. Wu Y. Li Z. Zhang Q.. (2022). Comparison of 7T and 3 T vessel wall MRI for the evaluation of intracranial aneurysm wall. Eur. Radiol. 32, 2384–2392. doi: 10.1007/s00330-021-08331-9, PMID: 34643780 PMC9207191

[ref14] Fiebach J. B. Galinovic I. (2014). MR imaging for acute stroke. Curr. Radiol. Rep. 2:53. doi: 10.1007/s40134-014-0053-0 PMC3972627 24707451

[ref15] Geurts L. J. Zwanenburg J. J. M. Klijn C. J. M. Luijten P. R. Biessels G. J. (2019). Higher Pulsatility in cerebral perforating arteries in patients with small vessel disease related stroke, a 7T MRI study. Stroke 50, 62–68. doi: 10.1161/STROKEAHA.118.022516, PMID: 30580730 PMC6314503

[ref16] Gilbert A. Detraz L. Alexandre P. L. Serfaty J. M. Desal H. Toquet C.. (2022). Magnetic resonance imaging quantitative T2* mapping to predict the red blood cell content of in vivo thrombi retrieved from patients with large vessel occlusions in acute ischemic stroke. Interv. Neuroradiol. 28, 523–530. doi: 10.1177/15910199211042473, PMID: 34559000 PMC9511618

[ref17] Hangel G. Niess E. Lazen P. Bednarik P. Bogner W. Strasser B. (2022). Emerging methods and applications of ultra-high field MR spectroscopic imaging in the human brain. Anal. Biochem. 638:114479. doi: 10.1016/j.ab.2021.114479 34838516

[ref18] Hansson B. Markenroth Bloch K. Owman T. Nilsson M. Lätt J. Olsrud J.. (2020). Subjectively reported effects experienced in an actively shielded 7T MRI: a large-scale study. J. Magn. Reson. Imaging 52, 1265–1276. doi: 10.1002/jmri.27139, PMID: 32196818

[ref19] Harteveld A. A. van der Kolk A. G. van der Worp H. B. Dieleman N. JJM Z. Luijten P. R.. (2017). Detecting intracranial vessel wall lesions with 7T-magnetic resonance imaging: patients with posterior circulation ischemia versus healthy controls. Stroke 48, 2601–2604. doi: 10.1161/STROKEAHA.117.017868 28701579

[ref20] Kang C. K. Park C. A. Park C. W. Lee Y. B. Cho Z. H. Kim Y. B. (2010). Lenticulostriate arteries in chronic stroke patients visualised by 7 T magnetic resonance angiography. Int. J. Stroke 5, 374–380. doi: 10.1111/j.1747-4949.2010.00464.x, PMID: 20854620

[ref21] Kaplan-Arabaci O. Acari A. Ciftci P. Gozuacik D. (2022). Glutamate scavenging as a Neuroreparative strategy in ischemic stroke. Front. Pharmacol. 13:866738. doi: 10.3389/fphar.2022.866738, PMID: 35401202 PMC8984161

[ref22] Klein V. Davids M. Schad L. R. Wald L. L. Guérin B. (2021). Investigating cardiac stimulation limits of MRI gradient coils using electromagnetic and electrophysiological simulations in human and canine body models. Magn. Reson. Med. 85, 1047–1061. doi: 10.1002/mrm.28472, PMID: 32812280 PMC7722025

[ref23] Koemans E. A. van Walderveen M. A. A. Voigt S. Rasing I. van Harten T. W. van Os J. A.. (2023). Subarachnoid CSF hyperintensities at 7Tesla FLAIR MRI: a novel marker in cerebral amyloid angiopathy. Neuroimage Clin. 38:103386. doi: 10.1016/j.nicl.2023.103386, PMID: 36989852 PMC10074985

[ref24] Leemans E. Cornelissen B. Sing M. L. C. Sprengers M. van den Berg R. Roos Y.. (2020). 7T versus 3T MR angiography to assess Unruptured intracranial aneurysms. J. Neuroimaging 30, 779–785. doi: 10.1111/jon.12772, PMID: 32857906 PMC7754498

[ref25] Lindenholz A. de Bresser J. van der Kolk A. G. van der Worp H. B. Witkamp T. D. Hendrikse J.. (2021). Intracranial atherosclerotic burden and cerebral parenchymal changes at 7T MRI in patients with transient ischemic attack or ischemic stroke. Front. Neurol. 12:637556. doi: 10.3389/fneur.2021.637556, PMID: 34025551 PMC8134532

[ref26] Lindenholz A. van der Kolk A. G. van der Schaaf I. C. van der Worp H. B. Harteveld A. A. Dieleman N.. (2020b). Intracranial atherosclerosis assessed with 7-T MRI: evaluation of patients with ischemic stroke or transient ischemic attack. Radiology 295, 162–170. doi: 10.1148/radiol.2020190643, PMID: 32013790

[ref27] Lindenholz A. van der Schaaf I. C. van der Kolk A. G. van der Worp H. B. Harteveld A. A. Kappelle L. J.. (2020a). MRI Vessel Wall imaging after intra-arterial treatment for acute ischemic stroke. AJNR Am. J. Neuroradiol. 41, 624–631. doi: 10.3174/ajnr.A6460, PMID: 32139427 PMC7144656

[ref28] Lucci C. Rissanen I. Takx R. A. P. van der Kolk A. G. Harteveld A. A. Dankbaar J. W.. (2024). Imaging of intracranial arterial disease: a comparison between MRI and unenhanced CT. Front Radiol. 4:1338418. doi: 10.3389/fradi.2024.1338418, PMID: 38426079 PMC10902099

[ref29] Madai V. I. von Samson-Himmelstjerna F. C. Bauer M. Stengl K. L. Mutke M. A. Tovar-Martinez E.. (2012). Sobesky J. Ultrahigh-field MRI in human ischemic stroke – a 7Tesla study. PLoS One 7:e37631. doi: 10.1371/journal.pone.0037631 22701525 PMC3365122

[ref30] Markenroth Bloch K. Poser B. A. (2021). “Chapter 35- benefits, challenges, and applications of ultra-high field magnetic resonance” in Advances in magnetic resonance technology and applications. eds. Choi I. Y. Jezzard P., vol. 4 (London, UK: Academic Press), 553–571.

[ref31] Miyazawa H. Natori T. Kameda H. Sasaki M. Ohba H. Narumi S.. (2019). Detecting lenticulostriate artery lesions in patients with acute ischemic stroke using high-resolution MRA at 7 T. Int. J. Stroke 14, 290–297. doi: 10.1177/1747493018806163, PMID: 30299228

[ref32] Mori F. Ishida F. Natori T. Miyazawa H. Kameda H. Harada T.. (2019). Computational fluid dynamics analysis of lateral striate arteries in acute ischemic stroke using 7T high-resolution magnetic resonance angiography. J. Stroke Cerebrovasc. Dis. 28:104339. doi: 10.1016/j.jstrokecerebrovasdis.2019.104339, PMID: 31451338

[ref33] Nicolo J. P. Moffat B. Wright D. K. Sinclair B. Neal A. Lui E.. (2021). 7T magnetic resonance imaging quantification of brain glutamate in acute Ischaemic stroke. J. Stroke 23, 281–284. doi: 10.5853/jos.2020.04784, PMID: 34102764 PMC8189859

[ref34] Nicolo J. P. O’Brien T. J. Kwan P. (2019). Role of cerebral glutamate in post-stroke epileptogenesis. Neuroimage Clin. 24:102069. doi: 10.1016/j.nicl.2019.102069 31795040 PMC6883323

[ref35] Okada T. Fujimoto K. Fushimi Y. Akasaka T. Thuy D. H. D. Shima A.. (2022). Neuroimaging at 7Tesla: a pictorial narrative review. Quant. Imaging Med. Surg. 12, 3406–3435. doi: 10.21037/qims-21-969, PMID: 35655840 PMC9131333

[ref36] Osuafor C. N. Rua C. Mackinnon A. D. Egle M. Benjamin P. Tozer D. J.. (2022). Visualisation of lenticulostriate arteries using contrast-enhanced time-of-flight magnetic resonance angiography at 7Tesla. Sci. Rep. 12:20306. doi: 10.1038/s41598-022-24832-z, PMID: 36434036 PMC9700841

[ref37] Özütemiz C. White M. Elvendahl W. Eryaman Y. Marjańska M. Metzger G. J.. (2023). Use of a commercial 7-T MRI scanner for clinical brain imaging: indications, protocols, challenges, and solutions-a single-center experience. AJR Am. J. Roentgenol. 221, 788–804. doi: 10.2214/AJR.23.29342, PMID: 37377363 PMC10825876

[ref38] Pan Z. Ma X. Dai E. Auerbach E. J. Guo H. Uğurbil K.. (2023). Reconstruction for 7T high-resolution whole-brain diffusion MRI using two-stage N/2 ghost correction and L1-SPIRiT without single-band reference. Magn. Reson. Med. 89, 1915–1930. doi: 10.1002/mrm.29573, PMID: 36594439 PMC9992311

[ref39] Perosa V. Priester A. Ziegler G. Cardenas-Blanco A. Dobisch L. Spallazzi M.. (2020). Hippocampal vascular reserve associated with cognitive performance and hippocampal volume. Brain 143, 622–634. doi: 10.1093/brain/awz383, PMID: 31994699 PMC7009470

[ref40] Pohmann R. Speck O. Scheffler K. (2016). Signal-to-noise ratio and MR tissue parameters in human brain imaging at 3, 7, and 9.4 tesla using current receive coil arrays. Magn. Reson. Med. 75, 801–809. doi: 10.1002/mrm.25677, PMID: 25820458

[ref41] Powers W. J. Rabinstein A. A. Ackerson T. Adeoye O. M. Bambakidis N. C. Becker K.. (2019). Guidelines for the early management of patients with acute ischemic stroke: 2019 update to the 2018 guidelines for the early management of acute ischemic stroke: a guideline for healthcare professionals from the American Heart Association/American Stroke Association. Stroke 50, E344–E418. doi: 10.1161/STR.0000000000000211 31662037

[ref42] Rados M. Mouthaan B. Barsi P. Carmichael D. Heckemann R. A. Kelemen A.. (2022). Diagnostic value of MRI in the presurgical evaluation of patients with epilepsy: influence of field strength and sequence selection: a systematic review and meta-analysis from the E-PILEPSY consortium. Epileptic Disord. 24, 323–342. doi: 10.1684/epd.2021.1399 34961746

[ref43] Regnery S. Knowles B. R. Paech D. Behl N. Meissner J. E. Windisch P.. (2019). High-resolution FLAIR MRI at 7Tesla for treatment planning in glioblastoma patients. Radiother. Oncol. 130, 180–184. doi: 10.1016/j.radonc.2018.08.002, PMID: 30177373

[ref44] Salmela M. B. Mortazavi S. Jagadeesan B. D. Broderick D. F. Burns J. Deshmukh T. K.. (2017). ACR appropriateness criteria ® cerebrovascular disease. J. Am. Coll. Radiol. 14, S34–S61. doi: 10.1016/j.jacr.2017.01.051 28473091

[ref45] Shafaat O. Sotoudeh H. Stroke imaging (Updated 2023 May 1). In: Stat Pearls. Treasure Island (FL): Stat Pearls Publishing; 2023 Jan-. Available online at: https://www.ncbi.nlm.nih.gov/books/NBK546635/

[ref46] Shaffer A. Kwok S. S. Naik A. Anderson A. T. Lam F. Wszalek T.. (2022). Ultra-high-field MRI in the diagnosis and Management of Gliomas: a systematic review. Front. Neurol. 13:857825. doi: 10.3389/fneur.2022.857825, PMID: 35449515 PMC9016277

[ref47] Shao X. Yan L. Ma S. J. Wang K. Wang D. J. J. (2021). High-resolution neurovascular imaging at 7T: arterial spin labeling perfusion, 4-dimensional MR angiography, and black blood MR imaging. Magn. Reson. Imaging Clin. N. Am. 29, 53–65. doi: 10.1016/j.mric.2020.09.003, PMID: 33237015 PMC7694883

[ref48] Shen Z. Xiang M. Chen C. Ding F. Wang Y. Shang C.. (2022). Glutamate excitotoxicity: potential therapeutic target for ischemic stroke. Biomed. Pharmacother. 151:113125. doi: 10.1016/j.biopha.2022.113125, PMID: 35609367

[ref49] Siemens Healthineers. (2025). Available online at: https://www.siemens-healthineers.com/magnetic-resonance-imaging/options-and-upgrades/clinical-applications/simultaneous-multi-slice

[ref50] Siemens Healthineers. Available online at: siemens-healthineers.com/terra.

[ref51] Spincemaille P. Anderson J. Wu G. Yang B. Fung M. Li K.. (2020). Quantitative susceptibility mapping: MRI at 7T versus 3T. J. Neuroimaging 30, 65–75. doi: 10.1111/jon.12669, PMID: 31625646 PMC6954973

[ref52] Stang A. (2010). Critical evaluation of the Newcastle-Ottawa scale for the assessment of the quality of nonrandomized studies in meta-analyses. Eur. J. Epidemiol. 25, 603–605. doi: 10.1007/s10654-010-9491-z, PMID: 20652370

[ref53] Suzuki T. Natori T. Sasaki M. Miyazawa H. Narumi S. Ito K.. (2021). Evaluating recanalization of relevant lenticulostriate arteries in acute ischemic stroke using high-resolution MRA at 7T. Int. J. Stroke 16, 1039–1046. doi: 10.1177/1747493019897868, PMID: 31955704

[ref54] Tan E. T. Hua Y. Fiveland E. W. Vermilyea M. E. Piel J. E. Park K. J.. (2020). Peripheral nerve stimulation limits of a high amplitude and slew rate magnetic field gradient coil for neuroimaging. Magn. Reson. Med. 83, 352–366. doi: 10.1002/mrm.27909, PMID: 31385628 PMC6778706

[ref55] Truong M. Markenroth Bloch K. Andersen M. Andsberg G. Töger J. Wassélius J. (2019). Subacute vessel wall imaging at 7-T MRI in post-thrombectomy stroke patients. Neuroradiology 61, 1145–1153. doi: 10.1007/s00234-019-02242-9, PMID: 31240344 PMC6754352

[ref56] Vachha B. Huang S. Y. (2021). MRI with ultrahigh field strength and high-performance gradients: challenges and opportunities for clinical neuroimaging at 7T and beyond. Eur. Radiol. Exp. 5:35. doi: 10.1186/s41747-021-00216-2, PMID: 34435246 PMC8387544

[ref57] van der Kolk A. G. Hendrikse J. Brundel M. Biessels G. J. Smit E. J. Visser F.. (2013). Multi-sequence whole-brain intracranial vessel wall imaging at 7.0 tesla. Eur. Radiol. 23, 2996–3004. doi: 10.1007/s00330-013-2905-z, PMID: 23736375

[ref58] Varela-Mattatall G. Dubovan P. I. Santini T. Gilbert K. M. Menon R. S. Baron C. A. (2023). Single-shot spiral diffusion-weighted imaging at 7T using expanded encoding with compressed sensing. Magnetic Res. Med. 90, 615–623. doi: 10.1002/mrm.29666, PMID: 37036384

[ref59] Wu X. Auerbach E. J. Vu A. T. Moeller S. Lenglet C. Schmitter S.. (2018). High resolution whole brain diffusion MRI at 7Tesla using radiofrequency parallel transmission. Magn. Reson. Med. 80, 1857–1870. doi: 10.1002/mrm.27189 29603381 PMC6107381

[ref60] Wu W. Poser B. A. Douaud G. Frost R. In M. H. Speck O.. (2016). High-resolution diffusion MRI at 7T using a three-dimensional multi-slab acquisition. NeuroImage 143, 1–14. doi: 10.1016/j.neuroimage.2016.08.054, PMID: 27570110 PMC5139985

[ref61] Yetisir F. Poser B. A. Grant P. E. Adalsteinsson E. Wald L. L. Guerin B. (2022). Parallel transmission 2D RARE imaging at 7T with transmit field inhomogeneity mitigation and local SAR control. Magn. Reson. Imaging 93, 87–96. doi: 10.1016/j.mri.2022.08.006, PMID: 35940379 PMC9789791

[ref62] Yoshimoto T. Inoue M. Yamagami H. Fujita K. Tanaka K. Ando D.. (2019). Use of diffusion-weighted imaging-Alberta stroke program early computed tomography score (DWI-ASPECTS) and ischemic Core volume to determine the malignant profile in acute stroke. J. Am. Heart Assoc. 8:e012558. doi: 10.1161/JAHA.119.012558, PMID: 31698986 PMC6915267

[ref63] Zhang C. Shi J. (2022). 7T MRI for intracranial Vessel Wall lesions and its associated neurological disorders: a systematic review. Brain Sci. 12:528. doi: 10.3390/brainsci12050528, PMID: 35624915 PMC9139315

